# Effect of Health Care Provider Delays on Short-Term Outcomes in Patients With Colorectal Cancer: Multicenter Population-Based Observational Study

**DOI:** 10.2196/15911

**Published:** 2020-07-17

**Authors:** Ahmed Abdulaal, Chanpreet Arhi, Paul Ziprin

**Affiliations:** 1 Imperial College London London United Kingdom

**Keywords:** surgery, cancer, colorectal, delay

## Abstract

**Background:**

The United Kingdom has lower survival figures for all types of cancers compared to many European countries despite similar national expenditures on health. This discrepancy may be linked to long diagnostic and treatment delays.

**Objective:**

The aim of this study was to determine whether delays experienced by patients with colorectal cancer (CRC) affect their survival.

**Methods:**

This observational study utilized the Somerset Cancer Register to identify patients with CRC who were diagnosed on the basis of positive histology findings. The effects of diagnostic and treatment delays and their subdivisions on outcomes were investigated using Cox proportional hazards regression. Kaplan-Meier plots were used to illustrate group differences.

**Results:**

A total of 648 patients (375 males, 57.9% males) were included in this study. We found that neither diagnostic delay nor treatment delay had an effect on the overall survival in patients with CRC (χ^2^_3_=1.5, *P*=.68; χ23=0.6, *P*=.90, respectively). Similarly, treatment delays did not affect the outcomes in patients with CRC (χ^2^_3_=5.5, *P*=.14). The initial Cox regression analysis showed that patients with CRC who had short diagnostic delays were less likely to die than those experiencing long delays (hazard ratio 0.165, 95% CI 0.044-0.616; *P*=.007). However, this result was nonsignificant following sensitivity analysis.

**Conclusions:**

Diagnostic and treatment delays had no effect on the survival of this cohort of patients with CRC. The utility of the 2-week wait referral system is therefore questioned. Timely screening with subsequent early referral and access to diagnostics may have a more beneficial effect.

## Introduction

Colorectal cancer (CRC) is the second most common cause of cancer-related deaths in the United Kingdom, and it accounted for 42,000 cases of cancer diagnoses in 2018 [1]. In fact, the United Kingdom has lower survival figures for all types of cancers than many European countries despite similar national expenditures on health [2]. The EUROCARE-4 study demonstrated that age-adjusted 5-year CRC mortality in the United Kingdom is significantly higher than that in the Nordic countries and Central Europe [2]. Abdel-Rahman et al [3] found that CRC accounted for the largest number of avoidable cancer-related deaths in the United Kingdom, with approximately 4090 avoidable cases.

Although surgery with curative intent is the preferred treatment modality for CRC [4], Gatta et al [5] found that only a small proportion of patients had undergone an elective procedure in the United Kingdom, usually owing to the advanced stage of cancer at diagnosis. A large proportion of patients with CRC are admitted as emergencies in the United Kingdom [6]. Emergency patients have a 1-year mortality that is ≥25% higher than patients who present through the screening and elective pathways [7]. The variability in the CRC survival is the greatest in the first year following diagnosis [8]; therefore, emergency patients may account in part for the higher 1-year mortality risk in the United Kingdom.

Thomson and Forman [9] demonstrated that patients with breast cancer who survive up to 1 year are more likely to survive up to 5 years. However, CRC is more complicated, as the 5-year conditional survival remains significantly worse for this cancer type [9]. This suggests that systematic delays such as delays in the referral, diagnosis, and treatment could have a constitutive effect on the long-term outcomes in patients in the United Kingdom and Europe [9]. Therefore, identifying and reducing the delays may lead to the detection of CRC at an early stage and diminish the proportion of emergency presentations, thereby eradicating the survival gap.

Previous studies have shown mixed results, while some studies have found no association [10], negative association [11], or “U-shaped” association [12] between delay and survival in patients with CRC. Many studies focus solely on the diagnostic interval [13] or consider general delays [14]. The aim of this study was to investigate the effect of diagnostic and treatment delays and their subdivisions on the survival of patients with CRC. We aimed to identify whether health care provider delays seen in the Imperial College Healthcare National Health Service Trust are related to the survival of patients with CRC. The hypothesis was that delays were associated with an increased risk of death.

## Methods

### Data Sources

Data were obtained from the Somerset Cancer Register, which is a database that collects wait times and outcomes data in line with the national database requirements [15]. Dataset collection was performed from January 2013 to March 2016.

### Study Population

A total of 5456 patients were investigated for CRC. Patients not diagnosed with CRC were excluded (n=4386). To ensure database validity, the patients’ sources of referral were examined. Of the excluded patients, 4118 (93.9%) patients within the first exclusion were referred through the 2-week wait pathway. In the United Kingdom, a 2-week wait referral is an urgent referral made by a patient’s general practitioner, wherein the patient should be seen within a 14-day period by a secondary care specialist. Such a referral should be made when a patient presents with symptoms that may indicate cancer. Of the 4118 patients with CRC, 246 were diagnosed through the 2-week wait pathway, representing a 5.9% conversion rate. This is in line with the 5.4% conversion rate that was reported for bowel cancer observed at the national level [16]. Patients whose date of diagnosis did not reflect a positive histology finding were excluded (Table 1, n=160). These groups were excluded owing to uncertain diagnoses. Utilizing the date of positive histology results as the date of diagnosis has been employed by another study [12].

**Figure 1:**
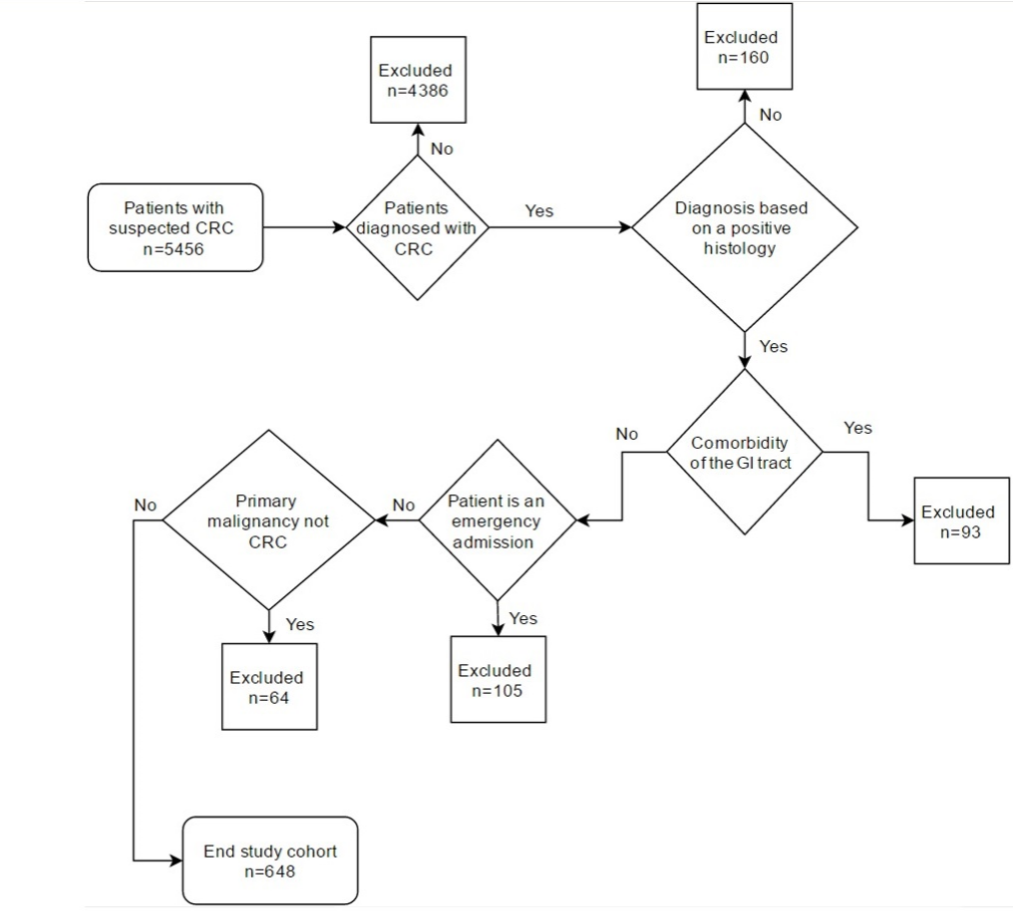
The algorithm used for patient inclusion. CRC: colorectal cancer; GI, gastrointestinal.

Patients with comorbid conditions of the gastrointestinal tract were excluded. This included patients with metastases from other primary cancers (n=11) or benign neoplasms (n=75). Patients with metastasis to the gastrointestinal tract may experience shorter diagnostic delays as a result of heightened physiological disturbance and yet exhibit worse outcomes [17], whereas those with benign neoplasms may exhibit a more insidious symptom development but a relatively favorable outcome [18,19]. Patients with inflammatory bowel disease were identified by searching multidisciplinary team reports for the following terms: colitis, proctitis, ulcerative, ulcerative colitis, Crohn(s), Crohn’s, and inflammatory bowel disease. Those with inflammatory bowel disease were excluded (n=7). Patients with inflammatory bowel disease represented 1.1% (7/648) of the cohort, which is in line with the expected prevalence of 1%-2% observed in all patients with CRC [20]. Patients who were referred following an emergency admission (n=105) were excluded. Emergency presentations typically experience shorter delays and worse 1-year and 5-year outcomes [7,21], which may produce a misleading negative association between the delay and the survival [22]. Patients diagnosed with malignancies of the small intestine, anus, or anal canal were excluded (n=64). The algorithm for patient inclusion is illustrated in Figure 1.

**Table 1:** Patient groups that were not diagnosed with colorectal cancer following a positive histology finding of a primary colorectal tumor (n=160).

Category of patients excluded	Patients, n (%)
A clinical diagnosis alone (patient symptomatology + a radiological investigation)	138 (86.2)
Diagnosis made after a positive serological tumor marker result	1 (0.6)
Unknown basis of diagnosis	1 (0.6)
Patients with an unrecorded basis of diagnosis	20 (12.5)

### Study Design

This was a multicenter population-based observational study. When assessing survival, other studies have demonstrated different trends based on the cancer type [23,24], and therefore, colon and rectal cancer cohorts were considered independently.

### Lead Time Bias

Patients included from the national bowel cancer screening program (n=92) were particularly susceptible to lead time bias. This bias occurs when outcomes are measured following diagnoses that reflect different starting points along the natural history of a cancer [25,26]. This may lead to a statistical extension in survival length without an actual increase in the duration of life for the patients detected through screening programs [14,27]. In order to account for the lead time, a correction by Duffy et al [28] was used, which estimates the additional follow-up time owing to earlier cancer detection. It assumes an exponential distribution of the sojourn time (E[s]) [29]—the interval in which a cancer is asymptomatic but can be detected by screening and is defined as *E(s) = (1-e^(-λt)^)/ λ)*, where t is the time at which a patient is last known to be alive and λ is the transition rate from preclinical to clinical cancer [28]. The transition rate is calculated as 1/mean sojourn time. Brenner et al [30] described age-specific and sex-specific estimates of the sojourn time for CRC. A weighted arithmetic mean sojourn time was calculated as 4.86; thus, λ=0.21. E(s) was subtracted from the observed survival time or time to the last known follow-up of patients referred through screening.

### Immortal Time Bias

Patients receiving treatment for their CRCs were necessarily alive between receiving a diagnosis and initiating treatment. This period is described as an immortal time, wherein the study outcome cannot occur [31]. Such patients may therefore have an artificial increase in their survival time if it is measured from the date of diagnosis, and this would introduce bias when analyzing the effect of the treatment delays on the study outcomes [31,32]. To obviate this bias, survival was measured from the date of the first treatment when considering the effect of the treatment delays. Survival was measured from the date of diagnosis when considering the diagnostic delays and overall delays.

### Study Variables

The effect of health care provider delay on survival was investigated. Survival was measured until death or censoring. Patients were censored at the last known live follow-up or at the end of the study period if no record of a follow-up is available; however, they were not recorded as deceased.

### Delay

**Figure 2:**
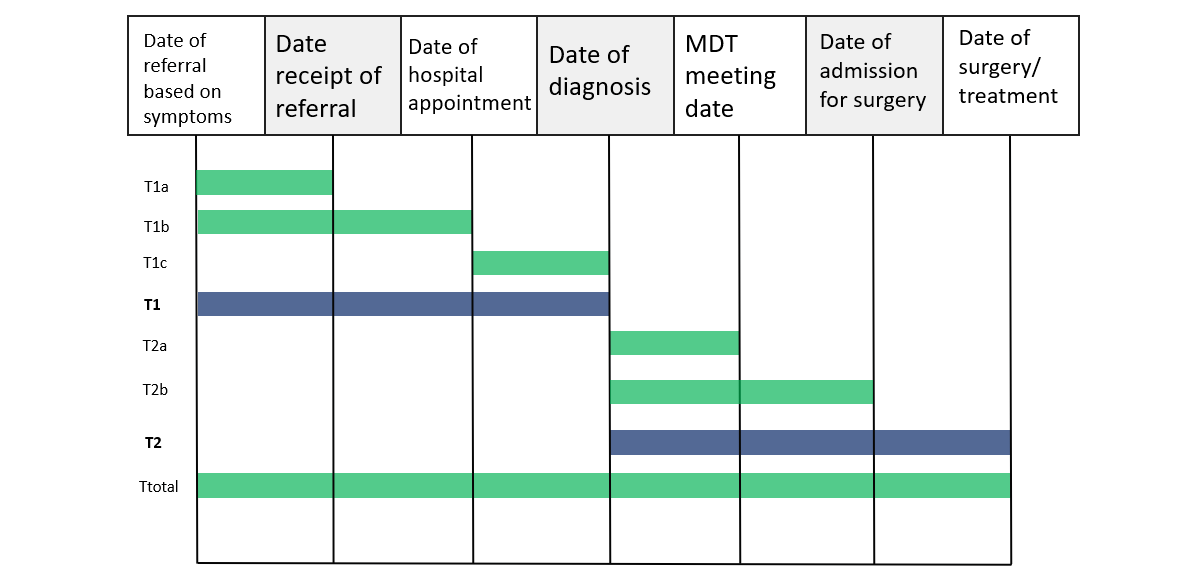
Representation of the delays and delay subdivisions considered for the analysis. T1: diagnostic delay; T1a: delay from referral based on symptoms to receipt of referral; T1b: referral delay; T1c: delay between hospital appointment and diagnosis; T2: treatment delay; T2a: delay between diagnosis and multidisciplinary team (MDT) meeting date; T2b: considered for those patients who received a surgical intervention; Ttotal: total delay from referral to surgery or treatment.

Delays were categorized into diagnostic and treatment delays. Delays and their subdivisions were analyzed separately as each delay type represents a discrete segment of the patient pathway [33]. Figure 2 illustrates all the delays considered in this analysis.

### Covariates

The covariates considered in this study were related to the patient demographics, including age, gender, and ethnicity. The data of the location, histology, grade, and stage of the tumor were also included. Patient performance status, which reflects the functional status of the patients [34], was also considered. Covariates that succeed diagnosis but may confound treatment delay and survival included treatment modality, intent (as categorized by synchronous insertion into the Somerset Cancer Register database at the time of treatment), and setting. These covariates were therefore included in the treatment delay models.

### Statistical Analysis

The median and IQR were calculated for diagnostic, referral, and treatment delays along with the delay quartiles. A survival analysis was conducted for all the delays and their subdivisions. Kaplan-Meier survival estimates were plotted for diagnostic and treatment delays by quartile. Group differences were analyzed using the log-rank test. The Cox proportional hazards regression analysis was used to investigate the effect of the covariates and to adjust for the confounding factors. To ensure the result validity, multiple sensitivity analyses were performed. Although deaths are regularly reported to the registry, diagnostic and treatment delay analyses were repeated for patients with a known live follow-up or death date. Next, all models were stratified by cancer stage, as stage may act as an intermediate factor between diagnostic delay and survival and it drives treatment regimens [14,23]. As suggested by previous researchers [13,35], analyses of diagnostic delays were repeated after excluding the covariates of tumor stage and grade to account for any confounding created by including them in the primary model. A *P* value of ≤.05 was considered statistically significant. SPSS statistics version 21 (IBM Corp) was used for the analysis.

## Results

### Study Sample

Of the 648 eligible patients, 375 were males (57.9%) and 272 were females (41.9%). Gender was not recorded for 1 patient (0.1%). The mean age was 69 years (range 29-96 years; 95% CI 67.8-70.2). There were 243 (37.5%) cases of rectal cancer and 405 (62.5%) cases of colon cancers. Of the 243 patients with rectal cancer, 30 (12.3%) died. Among the 405 patients with colon cancer, 38 (9.4%) died. The mean follow-up period for the patients with a known live follow-up was 383 days (95% CI 276.76-399.2). Patient characteristics are summarized in Table 2.

**Table 2:** Patient characteristics by cancer type (N=648).

Patient characteristics		Colon cancer cohort (N=405), n (%)	Rectal cancer cohort (N=243), n (%)
**Age (years)**			
	≤60	92 (22.7)	53 (21.8)
	61-65	52 (12.8)	32 (13.2)
	66-70	52 (12.8)	48 (19.8)
	71-75	57 (14.1)	46 (18.9)
	76-80	65 (16.0)	30 (12.3)
	81-84	51 (12.6)	12 (4.9)
	≥85	36 (8.8)	22 (9.1)
**Gender**			
	Male	229 (56.6)	146 (60.1)
	Female	176 (43.4)	96 (39.5)
	Unknown gender	0 (0)	1 (0.4)
**Race/ethnicity**			
	Caucasian	173 (42.7)	104 (42.8)
	Black	25 (6.2)	8 (3.3)
	Asian	20 (4.9)	8 (3.3)
	Mixed	2 (0.5)	2 (0.8)
	Other	41 (10.1)	26 (10.7)
	Unknown	144 (35.5)	95 (39.1)
**Cancer site^a^**			
	Proximal colon	169 (41.7)	N/A^b^
	Transverse colon	39 (9.6)	N/A
	Distal colon	186 (45.9)	N/A
	Unspecified colon	11 (2.7)	N/A
	Rectosigmoid junction	N/A	31 (12.8)
	Rectum	N/A	212 (87.2)
**Cancer stage^c^**			
	I	60 (14.8)	44 (18.1)
	II	65 (16.0)	43 (17.7)
	III	159 (39.3)	94 (38.7)
	IV	73 (18.0)	36 (14.8)
	Unknown	48 (11.9)	26 (10.7)
**Histology**			
	Adenocarcinoma	364 (89.9)	208 (85.6)
	Mucinous adenocarcinoma	16 (4.0)	7 (2.9)
	Signet ring cell carcinoma	2 (0.5)	0 (0)
	Neuroendocrine tumor	4 (1.0)	3 (1.2)
	Liposarcoma	1 (0.2)	0 (0)
	Other carcinoma	11 (2.7)	14 (5.8)
	Unknown histology	7 (1.7)	11 (4.5)
**Tumor differentiation**			
	Well differentiated (G1)	7 (1.7)	3 (1.2)
	Moderately differentiated (G2)	264 (65.2)	162 (66.6)
	Poorly differentiated (G3)	87 (21.5)	41 (16.9)
	Anaplastic (G4)	1 (0.2)	1 (0.4)
	Cannot be assessed (GX)	6 (1.5)	5 (2.1)
	Unknown differentiation	40 (9.9)	31 (12.8)
**Treatment type**			
	Active monitoring	4 (1.0)	10 (0.4)
	Chemotherapy	71 (17.5)	56 (23.0)
	Palliative care	15 (3.7)	7 (2.9)
	Surgery	292 (72.1)	140 (57.6)
	Radiotherapy	2 (0.5)	21 (8.6)
	Unknown treatment	21 (5.2)	18 (7.4)
**Treatment intent**			
	Adjuvant	21 (5.2)	7 (2.9)
	Curative	268 (66.2)	128 (52.6)
	Diagnostic	6 (1.5)	5 (2.1)
	Monitoring	4 (1.0)	1 (0.4)
	Neoadjuvant	6 (1.5)	7 (2.9)
	Palliative	30 (7.4)	25 (10.3)
	Radical/curative	3 (0.7)	17 (7.0)
	Unknown	67 (16.5)	53 (21.8)

^a^Proximal colorectal cancers are defined as cancers arising from the caecum up to and including the splenic flexure [36]. Cancers of the transverse colon are identified with the International Classification of Diseases for Oncology-10 code C184, which reflects “malignant neoplasms of the transverse colon.” Distal cancers are those arising in the descending (C186) or sigmoid (C187) colon.

^b^Not applicable.

^c^Dukes’ staging was reconciled with the TNM staging system as follows [37]: Dukes’ A or TNM stage T1-T2, N0, M0 = Stage I; Dukes’ B or TNM stage T3-T4, N0, M0 = Stage II; Dukes’ C or TNM stage T any size, N1, M0 = Stage III; Any metastasis = Stage IV.

### Diagnostic Delays

Diagnostic delays were calculated for 361 (89.1%) of the 405 patients with colon cancer and 216 (88.8%) of the 243 patients with rectal cancer. The median diagnostic delay was 34 days for both cancers (IQR 19-59 and 22-63 days, respectively). An analysis of the relationship between the cancer stage and diagnostic delay was performed. Diagnostic delays were right skewed and not normally distributed following the Kolmogorov-Smirnov test (*P*=.04); therefore, a Kruskal-Wallis H test was utilized. There was no correlation between diagnostic delay and cancer stage in the patients with colon cancer (*χ*^2^_4_=6.9, *P*=.14) or rectal cancer (*χ*^2^_4_=4.7, *P*=.32).

### Referral Delay

Referral delay was calculated for 390 (96.3%) of the 405 patients with colon cancer and 238 (97.9%) of the 243 patients with rectal cancer. The median referral delay was 10 days (IQR 4-15 days) for patients with colon cancer and 11 days (IQR 6-16 days) for patients with rectal cancer. The majority of the patients with colon cancer (285/390, 73.1%) and rectal cancer (172/238, 72.3%) experienced a referral delay of less than 2 weeks. However, 13.1% (51/390) of the patients with colon cancer and 13.4% (32/238) of the patients with rectal cancer experienced a referral delay of at least one month.

### Treatment Delays

Treatment delays were calculated for 327 (80.1%) of the 405 patients with colon cancer and 208 (85.6%) of the 243 patients with rectal cancer. The median treatment delay was 31 days (IQR 19-55 days) for patients with colon cancer and 42 days (IQR 27-106 days) for patients with rectal cancer. In all, 16.5% (54/327) of the patients with colon cancer and 11.5% (24/208) of the patients with rectal cancer experienced a treatment delay of <2 weeks. The majority of the patients with colon and rectal cancer experienced a treatment delay of ≥4 weeks (168/327, 51.4% and 142/208, 68.3%, respectively). Treatment delays displayed a similar skewness to diagnostic delays and were not significantly associated with cancer stage in either patients with colon or patients with rectal cancer (*χ*^2^_4_=8.6, *P*=.07 and *χ*^2^_4_=9.4, *P*=.054, respectively).

### Colon Cancer Delay and Survival

**Figure 3:**
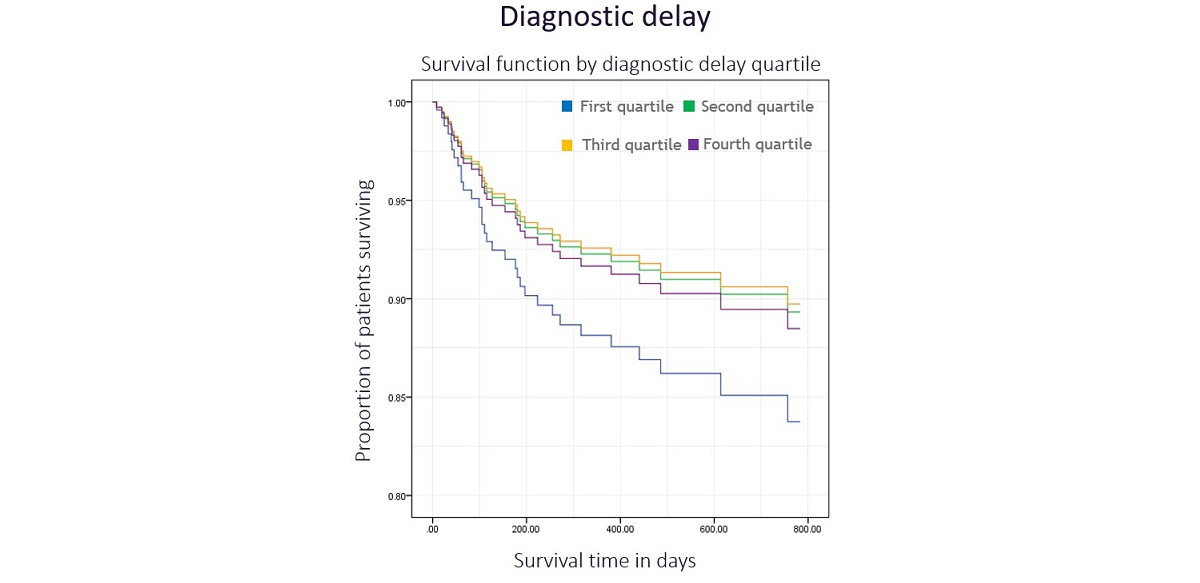
Kaplan-Meier plot illustrating the survival function by diagnostic delay quartile with time.

**Figure 4:**
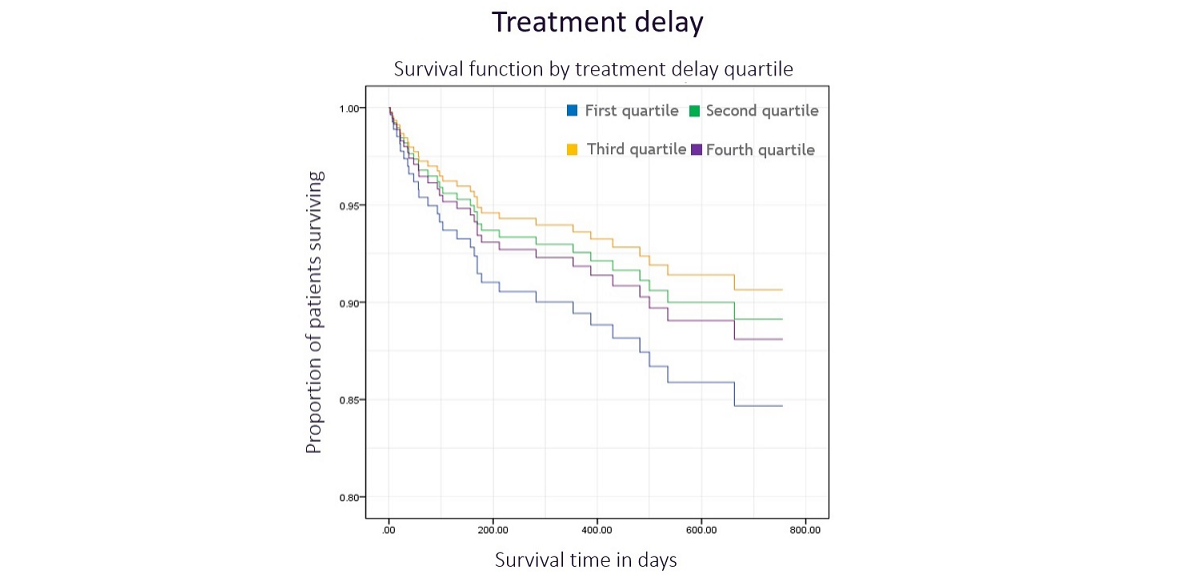
Kaplan-Meier plot illustrating the survival function by treatment delay quartile with time.

The log-rank test indicated no difference between long-term survival and diagnostic delay quartile (Figure 3, *χ*^2^_3_=1.5, *P*=.68). Diagnostic delay was a nonsignificant predictor of survival in the multivariate Cox regression model (*P*=.23). Additionally, there was no significant relationship between treatment delay quartile and survival in the log-rank test (Figure 4, *χ*^2^_3_=0.6, *P*=.90) or Cox regression model (*P*=.33). Tumor grade was an independent predictor of survival in both diagnostic and treatment delay models (*P*=.005 and *P*=.02, respectively), as was the tumor stage (*P*<.001 for both models).

### Rectal Cancer Delays and Survival

**Figure 5:**
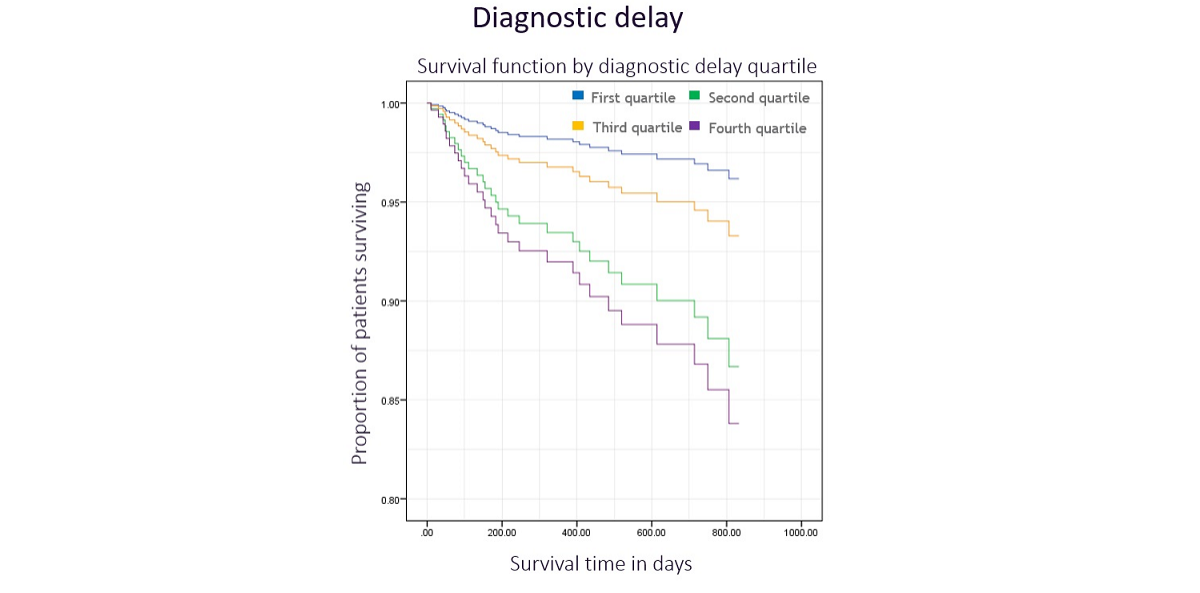
Kaplan-Meier plot illustrating the survival function by diagnostic delay quartile with time.

**Figure 6:**
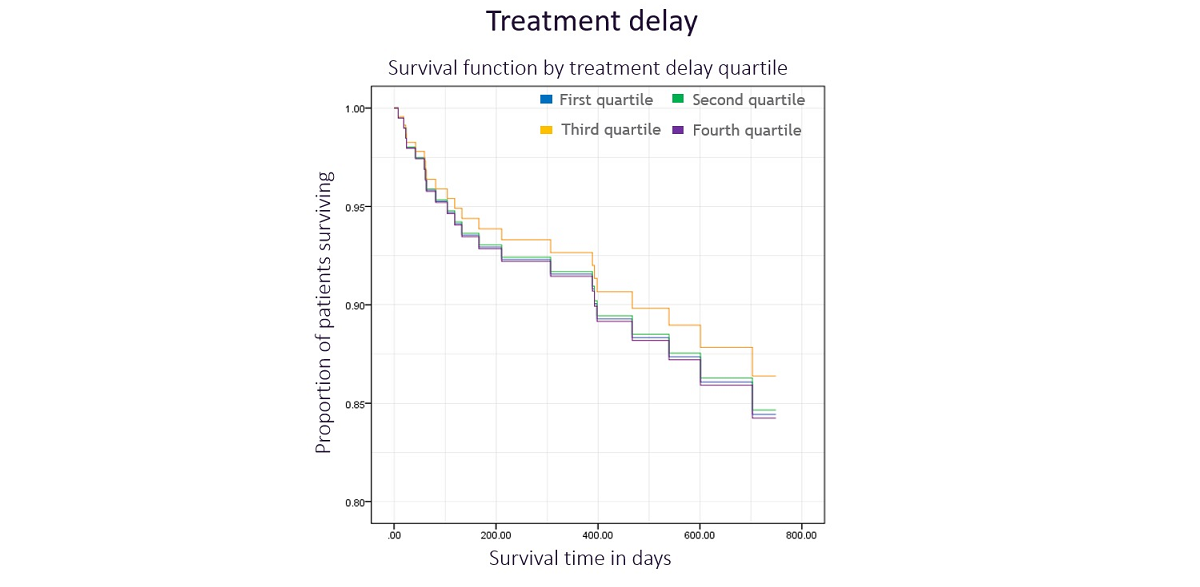
Kaplan-Meier plot illustrating the survival function by treatment delay quartile with time.

The relationship between diagnostic delay and survival in rectal cancer appears nonsignificant in the log-rank test (*χ*^2^_3_=5.5, *P*=.14). However, adjusting for covariates in the Cox regression model reveals a significant relationship between delay quartile and survival (*P*=.03). Patients with the shortest delays were significantly less likely to die than those with the longest delays (hazard ratio 0.165, 95% CI 0.044-0.616; *P*=.007). Figure 5 illustrates these results. Tumor stage remained significant (*P*=.04); however, tumor grade did not (*P*=.06). Treatment delays did not affect survival in either the log-rank test (*χ*^2^_3_=0.1, *P*=.99) or the Cox regression model (*P*=.98). Figure 6 illustrates the survival function by treatment delay quartile. None of the covariates analyzed were significant in this model, except for tumor stage, which achieved a borderline result (*P*=.053).

### Total Delay, Referral Delay, and Other Delay Subdivisions

In the analysis of total delay, treatment modality, intent, and setting were not included as covariates. Total delays were not significantly related to survival in either patients with colon cancer (*P*=.75) or in patients with rectal cancer (*P*=.35). Similarly, referral delays did not affect survival in either patients with colon cancer or patients with rectal cancer (*P*=.74 and *P*=.25, respectively). A summary of the bias and covariate adjusted analyses is shown in Table 3. However, the delay between the first hospital appointment and the date of diagnosis significantly affected the survival in patients with rectal cancer (Figure 2). Patients with the shortest delays were significantly less likely to die than those with the longest delays (hazard ratio 0.325, 95% CI 0.107-0.990; *P*=.048).

**Table 3:** Patient numbers and significance values for total delay, referral delay, and delay subdivision analyses.

Delay	Patients with colon cancer (N=405)		Patients with rectal cancer (N=243)	
	Patients, n (%)	*P* value	Patients, n (%)	*P* value
T1a^a^	399 (98.5)	.12	243 (100)	.64
T1b (referral delay)^b^	390 (96.3)	.74	237 (97.5)	.25
T1c^c^	344 (84.9)	.29	213 (87.6)	.048
T2a^d^	298 (73.5)	.56	187 (76.9)	.25
T2b (surgical patients only)^e^	237 (58.5)	.89	128 (52.7)	.69
Ttotal (total delay)^f^	375 (92.6)	.75	222 (91.3)	.35

^a^Delay between referral for symptoms and receipt of the referral by the hospital.

^b^Delay between referral based on symptoms and date of hospital appointment (referral delay).

^c^Delay between date of hospital appointment and date of diagnosis.

^d^Delay between date of diagnosis and multidisciplinary meeting date.

^e^Delay between date of diagnosis and admission for surgery.

^f^Delay between referral based on symptoms and date of the first surgical procedure or treatment (total delay).

### Sensitivity Analyses

There was good concordance between all models except for the effect of diagnostic delays on survival in patients with rectal cancer. A borderline result was obtained when censored patients were excluded (*P*=.052). Neither stratifying the models by cancer stage nor excluding covariates related to cancer behavior substantively altered the results. The results of the sensitivity analyses are shown in Table 4.

**Table 4:** Results of the sensitivity analyses.

Types of sensitivity analyses and delays		Patients with colon cancer (*P* value)	Patients with rectal cancer (*P* value)
**Sensitivity analysis 1: Excludes patients who do not have either a known follow-up date or date of death**			
	Diagnostic delay	.10	.05
	Treatment delay	.09	.34
**Sensitivity analysis 2: Stratifies colon and rectal cancer cohorts by cancer stage**			
	Diagnostic delay	.24	.01^a^
	Treatment delay	.12	.72
**Sensitivity analysis 3: Repeats analyses after excluding tumor stage and grade**			
	Diagnostic delay	.64	.03^b^
	Treatment delay	.70	.58

^a^The statistically significant relationship between diagnostic delay and survival in the rectal cancer cohort remained consistent when stratifying by cancer stage, where the first quartile group was significantly less likely to die than the fourth quartile group (hazard ratio 0.141, 95% CI 0.034-0.590; *P*=.01).

^b^When excluding tumor grade and stage, patients with the shortest delays were significantly less likely to die than those with the longest delays (hazard ratio 0.165, 95% CI 0.044-0.616, *P*=.03).

## Discussion

### Summary and Interpretation of Findings

This observational study investigated the relationship between health care provider delays and survival of patients with CRC. The median diagnostic delays were 34 days for both cancer types, while the median treatment delays for the patients with colon cancer and rectal cancer were 31 and 42 days, respectively. Contrary to the stated hypothesis, the health care provider delays had no effect on survival in this cohort.

Although longer diagnostic delays were associated with worse survival in the rectal cancer cohort, this relationship was statistically nonsignificant when restricting the analysis to patients with a known follow-up date or date of death. Further, although it is necessary to censor the patients who emigrate, are lost to follow-up, or for whom no date of death is recorded but who have not yet had a follow-up appointment, the nonsignificant result in this model may indicate that a disproportionately greater number of patients with shorter diagnostic delays were censored in the initial analysis. Considering this limitation, any conclusion regarding diagnostic delays in the rectal cancer cohort should be made tentatively.

Nonetheless, analysis of the delay subdivisions indicated that the delay between the first hospital appointment and diagnosis significantly affects survival. This may suggest that the effect on outcomes is due to unmeasured confounders relating to the nature of a patient’s diagnostic pathway. For example, frail patients may receive a computed tomography colonoscopy prior to an endoscopic procedure. These patients could experience longer diagnostic delays but are more likely to die. Future research should therefore adjust for the nature of the diagnostic testing performed, as this may confound the diagnostic interval and survival, thereby creating a spurious positive correlation between diagnostic delay and risk of death [12,13].

Previous studies have shown longer diagnostic delays in patients with colon cancer [38,39], which have been attributed to the symptoms being presented vaguely [40]. However, median diagnostic delays were the same for both cancers in this study. This may indicate a more homogenous group regarding presenting symptoms. Treatment delays were longer for patients with rectal cancer, and this is likely due to the higher incidence of neoadjuvant therapy [41], which requires oncological referral.

Risk of death increases for each stepwise progression in the cancer stage [42,43] and as expected, tumor stage was a significant predictor of survival in most models. Similarly, tumor grade was a significant covariate in many models; however, often with a smaller effect in increasing the hazard ratio of death. This may be due to the relative inconsequence of tumor grade in early-stage CRC. O’Connell et al [43] investigated the effect of tumor grade on survival by cancer stage and found a significant relationship between grade and survival in TNM stages II to IV but not stage I.

Previous literature has produced mixed results regarding the association between diagnostic delay and tumor stage. Ramos et al [44] found that delay was not significantly correlated with tumor stage. This finding was corroborated by several other researchers [45,46]—though not all the previous studies—with some researchers finding an inverse association between diagnostic delay and tumor stage [14,24,38]. Our study demonstrates no significant relationship between tumor stage and health care provider delays, contending the previously held notion that tumor stage is an intermediate factor between delay and survival [13].

### Comparison of the Main Findings with Previous Works

The paucity of evidence for a relationship between delay and survival in this study supports the results of previously published studies [22,47,48]. In a 2007 systematic review, 20 of the 26 studies found no association between delays and survival of patients with CRC [49]. Four studies found that longer delays were associated with favorable prognoses, with only 2 studies demonstrating an inverse relationship with worse outcomes. Studies that reported that longer delays lead to favorable outcomes likely fail to account for tumor aggressiveness either by restricting analysis to nonemergent cases [18] or by accounting for the confounding factor of the tumor grade [44,49].

There have also been various approaches to data analysis in this field. In a general practitioner–based study of 268 patients, a Danish group treated diagnostic delay as a continuous variable and conducted a restricted cubic spline regression analysis. This analysis revealed that patients who experienced >5 weeks of delay had a greater risk of death [13]. The study collected delay data retrospectively, making recall and information bias difficult to avoid. Additionally, they were unable to account for the tumor grade and considered colon and rectal cancers together.

A subsequent study of 958 patients with CRC by Murchie et al [35] also used restricted cubic spline regression analysis, which was adjusted for grade, symptoms, emergencies, and place of presentation. Furthermore, they used registry data and explored the relationship between delay and survival separately for colon and rectal cancers. They found no association between health care provider delay and the survival of patients with CRC.

Such conflicting results indicate that the relationship between health care provider delay and survival of patients with CRC remains uncertain [18]—an issue compounded by the ethical limitations of conducting a randomized control trial. Despite this, the evidence against the influence of delay on survival has remained consistent. However, it is important to note that median delays of 31-42 days for diagnosis and treatment in this study represented a relatively short period of time. It was therefore not possible to investigate the effect of lengthy delays on the survival of patients with CRC. Future research in settings wherein it is possible to measure the diagnostic delay from a patient’s subjective experience of symptoms or in areas with longer treatment delays may capture a relationship in the context of extended delays and survival.

Few studies have explored the effect of delays on postoperative outcomes such as readmission or complication rates. Psychosocial factors such as quality of life and anxiety are seldom assessed. Such outcomes should increasingly become the focus of future research.

### Context of the Findings

Timeliness and quality are not necessarily congruent and expediting the care of patients may be detrimental in certain circumstances. For example, McConnell et al [50] found that patients with CRC achieving a 4-week benchmark between diagnosis and surgery were less likely to have had preoperative staging. Although longer delays are undesirable, the 2-week wait pathway has not appreciably improved the outcomes and has increased the wait times for routine referrals, which remains the most common pathway for CRC diagnosis [51]. However, there is evidence that diagnosing CRC prior to symptom onset considerably improves survival. Annual occult blood tests reduce the 13-year cumulative mortality by 33% [52], and a single screening by sigmoidoscopy achieves similar results [53]. Public health initiatives should focus on improving compliance with screening programs, wherein prompt intervention improves outcomes.

### Strengths and Limitations

The Kaplan-Meier and Cox regression methods assume that censoring is independent of a patient’s risk of death. This may not have been the case, given the change in the significance between diagnostic delay and survival in the sensitivity analysis, which excluded censored patients. This suggests that the initial model underestimated the survival of patients with the shortest delays. However, others utilizing this technique have found the opposite, with censored patients being less likely to die, and therefore may have overestimated mortality in their analyses [12,54,55]. The magnitude and direction of this bias is therefore difficult to predict.

It was not possible to consider the initial presenting symptoms in this study. However, rectal bleeding has been associated with both poor [45] and improved [22] outcomes. Pruitt et al [14] stratified their cohort into 4 groups representing common presenting symptoms and found that this made no difference to their results. The effect of symptoms on survival is likely mediated by the cancer stage, which has been controlled for in this study.

There were also limitations associated with utilizing registry data. First, an analysis of patient delay was not possible, which is defined as the time between a patient noticing symptoms and presenting these symptoms to the general practitioner. However, patient delay data is often accrued through interviews or questionnaires, making recall bias difficult to avoid [18]. Even in prospective studies utilizing a structured interview format, there is often disagreement between patient responses and the clinical history [56,57]. Conclusions regarding patient delays should therefore be made cautiously. Secondly, survival should ideally be measured from the date of the first symptom presentation for diagnostic delay analysis [58,59]; however, this was not recorded in the Somerset Cancer Register. Finally, there was a short mean follow-up period of survival in this study, indicating that the conclusions are most relevant to 1-year survival rates. Continued follow-up of patients would allow for 5-year and 10-year survival trends to be analyzed in the future.

Despite these limitations, this study has several strengths. Registry data was entered synchronously with clinical practice, making this analysis resilient to recall bias [18]. Utilizing a population-based sample not restricted to those in tertiary care ensures more generalizable results. Unlike many previous studies, tumor aggressiveness and emergencies were controlled for, thereby minimizing the wait-time paradox. This study adjusted for several important biases and considered patients with colon cancer and rectal cancer separately. The Somerset Cancer Register data allowed an analysis of delay subdivisions, which ensured that important trends were not subsumed in a monotonic or a dichotomized delay model, while allowing clinically relevant conclusions about delays and their causes to be made. Finally, sensitivity analyses ensured the internal validity of the results.

### Conclusion

This observational study investigated the effect of health care delays on survival in patients with CRC. It is reasonable to conclude that the relatively short health care provider delays experienced by patients in the United Kingdom are not likely to affect the outcomes. Promoting effective screening programs should remain a high public health priority.

## Abbreviations

CRC: colorectal cancer
E(s): exponential distribution of the sojourn time
